# Critical appraisal of the European Union Scientific Committee on Health, Environmental and Emerging Risks (SCHEER) *Preliminary Opinion on electronic cigarettes*

**DOI:** 10.1186/s12954-021-00476-6

**Published:** 2021-03-10

**Authors:** Renée O’Leary, Riccardo Polosa, Giovanni Li Volti, the Center of Excellence for the Acceleration of Harm Reduction (CoEHAR), Salvatore Alaimo, Carmelina Daniela Anfuso, Ignazio Barbagallo, Francesco Basile, Sebastiano Battiato, Gaetano Bertino, Alberto Bianchi, Antonio G. Biondi, Maria Luisa Brandi, Emma Cacciola, Rossella R. Cacciola, Bruno Santi Cacopardo, Aldo E. Calogero, Maria Teresa Cambria, Davide Campagna, Filippo Caraci, Agatino Cariola, Massimo Caruso, Pasquale Caponnetto, Fabio Cibella, Maurizio Di Mauro, Santo Di Nuovo, Adriana Di Stefano, Filippo Drago, Salvatore Failla, Rosario Faraci, Salvatore Ferlito, Margherita Ferrante, Alfredo Ferro, Giancarlo A. Ferro, Francesco Frasca, Lucia Frittitta, Pio M. Furneri, Giovanni Gallo, Fabio Galvano, Antonio Gagliano, Giuseppe Grasso, Francesca Guarino, Antonino Gulino, Emmanuele A. Jannini, Sandro L. A. Vignera, Giuseppe Lazzarino, Antonio Longo, Gabriella Lupo, Mario Malerba, Luigi Marletta, Guido Nicolosi, Francesco Nocera, Gea Oliveri Conti, Rosalba Parenti, Alfredo Pulvirenti, Francesco Purrello, Francesco Rapisarda, Venerando Rapisarda, Michele Reibaldi, Renata Rizzo, Simone Ronsisvalle, Martino Ruggieri, Maria C. Santagati, Cristina Satriano, Laura Sciacca, Maria Salvina Signorelli, Marco Tatullo, Daniele Tibullo, Venera Tomaselli, Luca Zanoli, Agata Zappalà

**Affiliations:** 1 Center of Excellence for the Acceleration of Harm Reduction, Via S. Sofia, 89, 95123 Catania, Italy; 2 Catania, Italy; 3 Department of Clinical and Experimental Medicine, University of Catania, Catania, Italy; 4 Department of Biomedical and Biotechnological Sciences, University of Catania, Catania, Italy; 5 Department of Drug Sciences, University of Catania, Catania, Italy; 6 Department of General Surgery and Medical-Surgical Specialties, University of Catania, Catania, Italy; 7 Department of Mathematics and Computer Sciences, University of Catania, Catania, Italy; 8 National Observatory of Fragility Fractures, Catania, Italy; 9 Department of Medical, Surgical Sciences and Advanced Technologies, University of Catania, Catania, Italy; 10 Department of Biological, Geological and Environmental Sciences, University of Catania, Catania, Italy; 11 Department of Emergency Medicine, University of Catania Teaching Hospital Policlinico, Catania, Italy; 12 Department of Law Sciences, University of Catania, Catania, Italy; 13 Department of Educational Sciences, University of Catania, Catania, Italy; 14 Institute of Biomedicine and Molecular Immunology, National Research Council, Palermo, Italy; 15 Department of Chemical Sciences, University of Catania, Catania, Italy; 16 Department of Economics and Business, University of Catania, Catania, Italy; 17 Department of Electrical, Electronics and Computer Engineering, University of Catania, Catania, Italy; 18 Department of Systems Medicine, University of Rome Tor Vergata, Rome, Italy; 19 Department of Translational Biomedicine, University of Eastern Piedmont, Vercelli, Italy; 20 Department of Political and Social Sciences, University of Catania, Catania, Italy; 21 Technologica Research Institute, Marrelli Hospital, Crotone, Italy

**Keywords:** SCHEER, e-cigarettes, ENDS, Tobacco harm reduction, Gateway, Risk assessment, European Union Tobacco Products Directive

## Abstract

**Background:**

In preparation for the 2021 revision of the European Union Tobacco Products Directive, the Scientific Committee on Health, Environmental and Emerging Risks (SCHEER) has posted its *Preliminary Opinion on Electronic Cigarettes*. They concluded that e-cigarettes only achieve a sub-optimal level of protection of human health. In this paper, we provide evidence that the *Opinion’s* conclusions are not adequately backed up by scientific evidence and did not discuss the potential health benefits of using alternative combustion-free nicotine-containing products as substitute for tobacco cigarettes.

**Methods:**

Searches for articles were conducted in PubMed and by citation chasing in Google Scholar. Articles were also retrieved with a review of references in major publications. Primary data from World Health Organization surveys, the conclusions of reviews, and peer-reviewed non-industry studies were cited to address errors and omissions identified in the *Opinion*.

**Results:**

The *Opinion* omitted reporting on the individual and population health benefits of the substitution of e-cigarettes (ENDS) for cigarette smoking. Alternative hypotheses to the gateway theory were not evaluated. Its assessment of cardiovascular risk is contradicted by numerous reviews. It cites ever-use data that do not represent current patterns of use. It did not report non-nicotine use. It presented erroneous statements on trends in ENDS prevalence. It over-emphasized the role of flavours in youth ENDS initiation. It did not discuss cessation in sufficient length.

**Conclusions:**

For the delivery of a robust and comprehensive final report, the members of the Working Group of the Scientific Committee on Health, Environmental and Emerging Risks will need to consider (1) the potential health benefits of ENDS substitution for cigarette smoking, (2) alternative hypotheses and contradictory studies on the gateway effect, (3) its assessment of cardiovascular risk, (4) the measurements of frequency of use, (5) non-nicotine use, (6) the role of flavours, and (7) a fulsome discussion of cessation.

## Background

The European Union is in the process of revising the Tobacco Products Directive Article 28 to be submitted to the EU Parliament by 20 May 2021. The Scientific Committee on Health, Environmental and Emerging Risks (SCHEER) “Request for a scientific Opinion on Electronic cigarettes” [1] was mandated on February 7, 2019, and the *Preliminary Opinion on electronic cigarettes* [2] was posted online on September 23, 2020. An online comment process allowed a 1-month period for the submission of comments, with a limit of 3800 characters for each section of the *Opinion*. The Center of Excellence for the Acceleration of Harm Reduction (CoEHAR) at the University of Catania, Italy, approved the submission of comments on the *Opinion*. This paper adds additional comments to those we submitted to the online comments process and cites many additional studies and reviews. We refer to e-cigarettes as ENDS (electronic nicotine delivery systems), the terminology used by the World Health Organization (WHO). Their terminology could also be applied to another popular and very different product category, heat-not-burn or “heated tobacco product” that is electronic and delivers nicotine, but we have not included those products in this paper.

In reading the *Preliminary Opinion*, we were struck by its omission of any discussion of tobacco harm reduction, its limited discussion on the disputed gateway effect of youth ENDS use on cigarette initiation, and its appraisal of cardiovascular risks which is not in agreement with published reviews. A closer reading revealed its problematic reporting of the frequency of ENDS use, its decontextualized data on the role of flavours, and its omission of reporting on non-nicotine use.

Our critical appraisal of the *Opinion* was sharpened by the comments of other individuals and organizations. Dr. Karl E. Lund at the Norwegian Institute of Public Health circulated comments and references within an email network. Clive Bates posted two critiques [3, 4]. Christopher Snowdon at the EpiCenter, European Policy Information Center posted a response [5]. These publications provide additional criticisms of the *Opinion*.

Our critique seeks to address the errors and omissions we found in the *Preliminary Opinion*. Our critical appraisal is based on evidence from primary data (the WHO in particular), the conclusions of systematic and narrative reviews, and the findings of studies conducted by non-industry researchers.

## Methods

Multiple searches were conducted to obtain the primary data, reviews, and studies to support the critical appraisal of the *Opinion.* In addition to the searches, we referenced two authoritative reviews on ENDS.

We used three primary sources of data. To address the *Opinion’s* statements on renormalization and a possible impact of ENDS use on smoking cessation trends, we extracted primary data from the most recent WHO reports [6, 7]. For the prevalence of ENDS use, we extracted data from the Passport database by EUROMONITOR [8], an established consumer products research organization.

We searched PubMed for reviews and studies on the subject areas of cessation, cardiovascular risks, the gateway effect, and the impact of flavours on youth initiation and adult cessation. Searches were conducted with the terms “e-cigarettes” AND “review” AND [subject keyword] in the title/abstract field. Subject keywords: cessation, cardiovascular, youth (OR student OR adolescent), biomarkers, prevalence. These searches were performed individually for each subject keyword. Another set of searches was conducted with the keywords “e-cigarette” AND [individual EU member state] in the title/abstract field.

Studies and reviews were also identified from published authoritative key sources including the *Public Health Consequences of E-Cigarettes* [9] and the *Report on the Scientific Basis of Tobacco Product Regulation: Seventh Report of a WHO Study Group* [10]. We also referenced studies from *Vapour Products/E-Cigarettes: Claims and Evidence* [11] and the monograph *Clearing the Air: A systematic review on the harms and benefits of e-cigarettes and vapour devices* [12]. Finally, reviews and studies in the *Opinion* were searched for additional findings. All reviews and studies retrieved were citation searched (snowball search) in Google Scholar to identify additional publications.

The results of the search were reviewed for best fit for our comments on the *Opinion* as the comment sections were strictly limited in size. Studies of EU member states were prioritized over those from the US due to the substantial differences between their ENDS regulations and their commercial markets. Nevertheless, for some subjects only US research was available or provided major studies. These data, reviews, and studies form the basis of the critical appraisal that follows.

## Omitted from the terms of reference: tobacco harm reduction

The background section of the *Request for Scientific Opinion* included the role of ENDS in harm reduction. Yet the evaluation of harm reduction was not specifically stated as an item in the Terms of Reference, obliquely referring to “their use.” This wording in the *Request* resulted, intentionally or unintentionally, in the omission of the key role of ENDS in tobacco harm reduction. The potential effects of ENDS on individual and public health are the subject of a tremendous number of studies and medical association position statements. The *Opinion* is seriously incomplete without an evaluation of ENDS for tobacco harm reduction. We present evidence on how ENDS have the potential to reduce the risks of smoking for the substantial number of EU citizens who smoke.

### Not quitting

The assumption that people who smoke want to quit is drawn from self-reports in surveys, but it constitutes a vague wish. On the other hand, when asked directly about quitting activities, a staggeringly high number of EU adults who smoke have no intention to quit [13], see Table 1.

**Table 1:** EU smoking adults with no quit intentions, EUREST-PLUS ITC survey 2016. *Source*: [13]

Member state	No intention to quit (%)
Germany	42.4
Greece	59.5
Hungary	68.1
Netherlands	18.9
Poland	58.7
Romania	46.4
Spain	63.5

Furthermore, for those that wish to quit, smoking cessation rates are very low, from 3 to 12%, and relapse rates are very high, from 75 to 80% in the first 6 months and 30%–40% even after 1 year of abstinence [see studies cited in 14]. Quitting is not a single event but a dynamic process, and relapse is a common component of this process. While international guidelines place great emphasis on relapse prevention, very little can be offered to help those who have relapsed [15]. This challenge calls for innovative and effective alternatives for relapse prevention, including the use of ENDS.

### Reduced emissions compared to cigarettes

For those who do not make the effort to quit and for those who cannot quit or remain abstinent, ENDS may provide an alternative to smoking. It is generally accepted that ENDS have substantially lower and fewer toxic emissions than cigarettes. The National Academies of Science, Engineering, and Medicine state “There is substantial evidence that except for nicotine, under typical conditions of use, exposure to potentially toxic substances from e-cigarettes is significantly lower compared with combustible tobacco cigarettes” [9, p. 6]. Many toxic substances in cigarettes are not emitted by ENDS, as clearly illustrated in the research [16] that did not detect 61 of 79 compounds present in tobacco smoke. Testing on ENDS liquids compared to cigarettes (mL liquid, gram tobacco) found that nitrosamines, a major source of negative health effects and cancer, were over 400 times lower in ENDS liquids than cigarettes, and nitrate was over 1300 times lower and phenols were 1200 times lower than cigarette smoke [17]. The findings of lower emissions in ENDS compared to tobacco smoke were corroborated in the review by the European Respiratory Society [18] and other reviews [19, 20]. Stephens [21] calculated the cancer potency of ENDS emissions to have 0.004 of the relative lifetime cancer risk of tobacco smoke. While major assumptions and guesswork are required to translate reductions in emissions into an estimate of actual health risks, it is impossible to believe that the orders-of-magnitude differences do not represent enormously lower risk. The *Opinion* does not compare the emission levels of ENDS vapour with tobacco smoke.

The UK regulatory organization, Committee on Toxicity of Chemicals in Food, Consumer Products and the Environment conducted an extensive and systematic review on the potential toxicological risks of ENDS [22]. Their conclusion was that:

The use of E(N)NDS products, produced according to appropriate manufacturing standards and used as recommended, as a replacement for CC [cigarette] smoking, is likely to be associated with a reduction in overall risk of adverse health effects, although the magnitude of the decrease will depend on the effect in question. (p. 28)

### Biomarker evidence

Except for one study on nicotine exposure, the *Opinion* does not present biomarker studies. Adding biomarker data to the *Opinion* is vital as research has found that exposure levels for a number of toxins are similar between people who use ENDS and people who have never smoked. Biomarker data is relevant evidence for showing reductions in toxicant exposures from ENDS compared to tobacco smoke to demonstrate tobacco harm reduction. The following studies represent some of the many biomarker studies that observed a substantial reduction in levels of toxins in the bodies of the participants who substituted ENDS for tobacco smoking.

The *Opinion* considered metal exposures, but did not cite a study [23] that reviewed blood lead (*N* = 1899) and urinary cadmium, barium, and antimony (*N* = 1302) test data in the 2015–2016 US National Health and Nutrition Examination Survey (NHANES). There were no significant differences in the levels of exposure to metals between participants who had never used ENDS and participants who were current or former ENDS users. The researchers concluded that ENDS were not a source of exposure to these heavy metals.

In a biomarker study funded by the US National Cancer Institute [24] gave urine tests to 28 participants who were using ENDS and who had quit smoking for a minimum of 2 months. The participants had significantly lower biomarkers of exposure compared to those who smoked cigarettes. Their 1-HOP levels (exposure to polycyclic aromatic hydrocarbons) were similar to people who did not smoke. The study found significantly lower levels of metabolites in those using ENDS compared to those smoking cigarettes. These were:

- 1-hydroxypyrene/1-HOP (a marker for polycyclic aromatic hydrocarbons [PAH])
- 4-(methylnitrosamino)-1-(3-pyridyl)-1-butanol and its glucuronides/total NNAL (a marker for nicotine toxin)
- 3-hydroxypropylmercapturic acid/3-HPMA (a marker for acrolein)
- 2-hydroxypropylmercapturic acid/2-HPMA (a marker for propylene oxide)
- 3-hydroxy-1-methylpropylmercapturic acid/HMPMA (a marker for crotonaldehyde)
- *S*-phenylmercapturic acid/ SPMA (a marker for benzene).

The researchers concluded that:

levels of a suite of urinary toxicant and carcinogen metabolites were significantly lower in e-cigarette users than in cigarette smokers. These results suggest that e-cigarette use may be safer than cigarette smoking, at least with respect to the compounds studied here, which represent typical carcinogens and toxicants believed to be involved in causing cancer in cigarette smokers. (p. 708).

A clinical study [25] examined the effects of 4 weeks of ENDS substitution with 33 participants who smoked. The mean 3-HPMA levels (acrolein) decreased 79% for those exclusively using ENDS and 60% for those using both ENDS and cigarette. Carbon monoxide levels decreased by 80% in for participants with exclusive ENDS use and 52% in those using both ENDS and cigarettes. Reduction of carbon monoxide levels to within normal limits has been reported soon after switching from conventional cigarette smoking to exclusive ENDS use in other studies [26–28].

A before and after study [29] tested 20 Polish adults who smoked cigarette users for biomarkers of exposure after 2 weeks, with half of the participants substituting ENDS and half continuing to smoke. Significant reductions in exposure levels were detected for many toxicants in the participants substituting ENDS, as listed in Table 2.

**Table 2:** Significant reductions in toxin exposures with short-term ENDS substitution. *Source*: [29, Supplemental Table 3]

Toxicant	Significant reduction *p* < 0.05 (%)
NNK Nitrosamine ketone	64
Ethylene oxide	61
1,3-Butadiene	84
Crotonaldehyde	67
Acrolein	56
Benzene	76
Acrylamide	57
Acrylonitrile	79
Propylene oxide	53
PAH polycyclic aromatic hydrocarbon Biomarker 1-Hydroxyfluorene	58
PAH polycyclic aromatic hydrocarbon Biomarker 3-Hydroxyfluorene	34

The substantial and significant reduction in 1,3-butadiene is particularly noteworthy as it is the greatest source of cancer risk in tobacco smoke [30]. Goniewicz et al. conclude that “e-cigarettes may effectively reduce exposure to toxic and carcinogenic substances among smokers who switched to these products” (p. 165).

In a 4-week observational study conducted with 40 adults who were smoking cigarettes who added or substituted ENDS use and biomarker levels for exposure to NNAL, benzene, and acrylonitrile were significantly reduced in all participants [31]. Participants reporting exclusive ENDS use for at least 2 weeks exhibited additional significant reductions in metabolite levels of ethylene oxide and acrylamide. Participants exclusively using ENDS had reductions in acrolein levels bringing them into the range of persons who do not smoke.

While significant reductions in biomarkers of exposure are not evidence of an absence of risk, these studies (and industry studies not cited) demonstrate that exposures to toxicants are substantially and significantly lower for those using ENDS compared to those who smoke tobacco. People who smoke can substantially reduce their exposure to known toxicants by substituting ENDS for smoking tobacco, even when it is not complete substitution.

### Individual and population health benefits

We are not aware of any studies showing a negative impact on the health of individuals who substitute ENDS for tobacco smoking. Although concern has been raised about the long-term health impacts of ENDS, a handful of short-term and long-term clinical trials have demonstrated health benefits from ENDS substitution. It is regrettable that there are so few clinical trials on ENDS health effects outside of cessation, although smoking cessation has known health benefits. Complete ENDS substitution for smoking may well prove over time to substantially lessen the risks of continued smoking.

In a 12-month assessment of the impact of ENDS use on blood pressure in 89 patients with hypertension, consistent and clinically significant improvement in systolic and diastolic BP as well as in BP control was observed in those who switched to regular ENDS use [32]. These findings are in agreement with the 8.8 mmHg reduction in systolic BP at 12 months in a prospective randomized control trial looking at the effect of smoking cessation by using ENDS in subjects with high BP at baseline [33].

A randomized controlled trial (*N* = 114) [34] demonstrated that 4 weeks of ENDS substitution for smoking resulted in significant improvements in flow-mediated dilation and decreases in vascular stiffness compared to the cigarette user arm, indicating a reduced risk for cardiovascular disease.

The respiratory health effects of ENDS have been addressed in two recent review articles, and their conclusions are conflicting [35, 36]. Both these reviews cite in vitro and in vivo studies, but findings from human studies support the view that ENDS use shows no evidence of health harms and even health improvements in patients with chronic obstructive pulmonary disease (COPD) or asthma.

In a small study of participants who used ENDS daily and who had never smoked, no noticeable changes in health outcomes were detected over the 3.5-year observation period [37]. Daily usage of ENDS caused no significant changes in measures of lung function, respiratory symptoms and lung inflammation, and no significant structural abnormalities could be identified on high-resolution computerized tomography (HRCT) of the lungs.

A 5-year follow-up clinical study (assessments at 12, 24, 48, and 60 months) of patients with COPD compared 19 patients who completely or partially substituted ENDS for smoking to 20 controls who smoked [38]. Switching to ENDS contributed to a rapid improvement in cardiorespiratory health, with better quality of life, improved exercise tolerance, and an approximately 50% reduction in COPD exacerbations. Of note, these health gains were consistent throughout the 5-year follow-up.

A study [39] evaluated participants with asthma who used ENDS exclusively and who had stopped smoking. In one section of the study, a web survey (*N* = 382), 91.6% survey participants self-reported no worsening of symptoms from ENDS use. The second part of the research conducted clinical testing of 10 participants with asthma who used ENDS at baseline, 3 months, and 6 months. The participants experienced a significant increase in asthma symptom control and improvements in the Asthma Quality of Life Questionnaire (AQLQ) scores over the course of the study.

In a 2-year prospective clinical study, ENDS use consistently improved objective and subjective asthma outcomes [40]. ENDS use was well tolerated, and exposure to e-liquid aerosol in this vulnerable population did not trigger any asthma attacks.

A positive impact of ENDS substitution on population health has been predicted by population-based models. Prochaska and Benowitz, leading expert researchers on tobacco, stated:

“While e-cigarettes may have adverse effects on respiratory health and possibly other diseases, the harm is generally accepted to be much less than that of cigarette smoking. Thus, if smokers were to switch completely to e-cigarettes, then smoking-related disease is predicted to decrease substantially. Population-based models of the impact of e-cigarette use predict an overall health benefit” [41, pp. 17–18].

The WHO Study Group on Tobacco Product Regulation concurs, “The available evidence indicates a possible positive effect of ENDS on population health, particularly if appropriate ENDS regulation is enacted to maximize their benefits and minimize their risks” [10, p. 60]. In addition to health benefits, economic benefits would result from reductions in direct costs for healthcare and indirect costs from lost productivity and disability benefits [42]. The potential benefits of ENDS substitution for tobacco smoking on individual and population health certainly merit its inclusion in the final report.

## The gateway effect

### Common liabilities, an alternative hypothesis

The Terms of Reference specified an examination of a gateway effect. The gateway hypothesis is not the only explanation for the observed correlation between youth ENDS and tobacco smoking prevalence [9, 10, 43]. The common liabilities theory posits that a “common latent propensity to risky behaviour” leads to concurrent tobacco smoking and ENDS use [10, p. 57]. A review by the European Respiratory Society stated that shared risk factors are “likely alternative explanations supported by the literature” [18, p. 14]. Hammond et al. [44] in a 1-year longitudinal cohort study (Canada, *N* = 19,130) concluded that “it is highly plausible that ‘common factors’ account for a substantial proportion of increased cigarette-smoking initiation among e-cigarette users” (p. E1135). The common liabilities hypothesis that psychosocial factors explain poly-substance use by youth should be evaluated as an alternative explanation to a gateway effect.

In a recent editorial in the *American Journal of Public Health* [45], the gateway theory was labelled a “common deception.” Review teams are more sanguine, but unconvinced of a gateway hypothesis. A narrative review by [46] on youth ENDS use stated that there is no strong evidence supporting the gateway hypothesis. A systematic review and meta-analysis [47] on adolescent ENDS use and smoking initiation reached the conclusion that “it is not clear how much of the relationship is causal (gateway effect) or is due to common liability…there is much less conclusive evidence for a gateway effect” (p. 13).

While no definitive explanation has been reached on why youth ENDS ever-users have a higher prevalence rate of smoking than those who never used ENDS, almost all of the studies used US data. Considering this, we think it is important to note the evidence from a major study of French youth, the Escapad Survey conducted with government compulsory participation for all 17-year-olds [48]. In the 2017 survey (*n* = 17,862 ever smokers) showed that youth who ever-used ENDS were less likely to smoke cigarettes daily (RR = 0.62, CI 0.60–0.64) than youth who smoked and who had never tried ENDS. Youth who tried ENDS first before ever-smoking were less likely to smoke cigarettes daily (RR = 0.76, CI 0.66–0.89). Chyderiotis et al. observed that these findings were “in contradiction with the gateway hypothesis” [48, p. 5]. Of course, no one study can prove or disprove a gateway effect, but this cross-sectional study is one of the very few studies available investigating a gateway effect among youth from an EU member state.

## Inaccurate statements on EU prevalence trends

### Renormalization

The *Opinion* alludes to a “resurgence of cigarette smoking” suggesting a renormalization of smoking. This is not happening, as clearly shown by the accelerated decline in smoking prevalence in EU member states, some of which have the highest prevalence of ENDS users. Changes in the trends of smoking prevalence are the best indicator of the absence or presence of renormalization [49]. Based on WHO data [6, 7], between 2016 and 2018, 24 of 27 EU member states experienced declines in the prevalence of cigarette use for the population 15 years old and older. Seven member states had cigarette prevalence declines of 6% or better and three member states had declines over 10% during the 2-year period. See Table 3.

**Table 3:** Declines/increases in cigarette smoking prevalence in EU member states, 2016–2018. *Source* World Health Organization [6, Table A1.4], World Health Organization [7, Table A.1.3]

Member state	Cigarette smoking prevalence 2018	Cigarette smoking prevalence 2016	% Change in prevalence (%)
Austria	24.1	25.1	− 4.0
Belgium	21.4	23.8	− 10.1
Bulgaria	31.4	30.0	4.6
Croatia	29.6	29.9	− 1.0
Cyprus	29.1	29.4	− 1.0
Czechia	24.7	26.4	− 6.5
Denmark	16.9	17.7	− 4.5
Estonia	24.5	25.5	− 3.9
Finland	15.3	15.5	− 1.2
France	27.0	26.0	3.8
Germany	22.6	24.4	− 7.8
Greece	33.1	35.8	− 7.6
Hungary	25.8	26.0	− 0.8
Ireland	20.0	20.3	− 1.5
Italy	20.8	21.2	− 1.9
Latvia	28.9	30.0	− 5.7
Lithuania	21.6	23.2	− 6.9
Luxembourg	18.9	20.1	− 6.0
Malta	20.6	21.1	− 2.4
Netherlands	19.1	21.3	− 10.4
Poland	23.7	25.2	− 6.0
Portugal	20.4	17.6	15.9
Romania	21.1	24.3	− 13.1
Slovakia	25.3	24.1	4.9
Slovenia	18.5	18.8	− 1.5
Spain	22.8	23.8	− 4.2
Sweden	12.1	13.3	− 9.0

### ENDS prevalence trends

The highest ENDS prevalences are between 4.1 and 5.7% in eight member states, while the prevalence is under 2% in 13 member states. Nor are EU prevalences “increasingly rising” as stated in the *Opinion*, and in fact, they have been relatively stable from 2017 to 2019. During this period, three member states had no increase in the prevalence and seven member states had an increase of 0.2 percentage points or less. Only two member states had an increase in the prevalence of 1.0% or higher, the Netherlands with 1.0% and Portugal with 1.3% [8]. See Table 4.

**Table 4:** Prevalence of Adult ENDS use, EU Member States, 2017 and 2019. *Source*: [8 Passport Database, Adult Smokers—Historical Data, Vapour Products]

Member state	ENDS prevalence 2019	ENDS prevalence 2017
Austria	1.6	0.9
Belgium	4.8	4.1
Bulgaria	1.1	1.1
Croatia	1.7	0.8
Cyprus	NR	NR
Czechia	5.7	5.6
Denmark	5.1	4.6
Estonia	1.6	1.9
Finland	2.0	1.9
France	4.4	4.2
Germany	5.4	5.2
Greece	2.6	2.4
Hungary	1.9	2.4
Ireland	5.5	5.3
Italy	1.7	1.4
Latvia	1.2	0.9
Lithuania	1.9	1.1
Luxembourg	NR	NR
Malta	NR	NR
Netherlands	4.1	3.1
Poland	5.4	5.4
Portugal	2.8	1.5
Romania	3.5	3.5
Slovakia	2.1	1.8
Slovenia	0.9	0.8
Spain	1.7	1.2
Sweden	1.0	1.0

*NR* not reported

The Working Group should not imply that ENDS may cause a resurgence of cigarette use because cigarette use had declined, often substantially, in almost every EU member state. Consumer data on the size of the market for adults purchasing ENDS contradict the statement that the prevalence of ENDS use among EU adults is rising quickly. In many EU member states, ENDS usage prevalence has been relatively stable with only a very small rise, and in some member states no rise, between 2017 and 2019.

#### Youth prevalence

For data on the prevalence of youth use, the ESPAD report 2019, the *European School Survey Project on Alcohol and Other Drugs*, was published subsequent to the *Opinion*. Its findings should be included in the final report. Some prevalence data in the *Opinion* are from 2015 and earlier and so have a limited value for reporting current prevalences. Much of the prevalence data in the *Opinion* are for ever-use, a measurement that grossly over-represents the actual number of ENDS users, as discussed below.

## Mismeasurements in exposure assessments

### Frequency of use

The *Opinion* frequently cites ever-use data. Ever-use is a problematic measurement that captures a substantial number of persons who tried ENDS only one time and substantially overestimates actual usage. Indeed, it does not even track it, given that within a cohort ever-use will always increase over time even if usage prevalence drops.

Ever-use data on youth is unreliable for exposure assessment “as ever use can include using an e-cigarette once across the lifetime, the extent of increased nicotine exposure as a result of ever e-cigarette use is unclear” [50, p. 616]. Data from the Global Youth Tobacco Survey (GYTS) showed that 27–55% (varying by country) of EU youth who ever tried ENDS did so on only one occasion [51]. See Table 5. For Italian youth (15–19 years old), the ESPAD® Italia [European School Survey Project on Alcohol and other Drugs] 2017 survey found that over 70% of youth who reported using ENDS had done so only one to nine times [52, supplementary material].

**Table 5:** Prevalence of one occasion ENDS use in youth ever-users. *Source*: World Health Organization [51] “Electronic cigarette smoked all life”

Member state	GYTS year	% ever-users tried only one
Bulgaria	2015	42.6%
Croatia	2016	36.9%
Finland	2012	51.7% once or twice
Malta	2017	40.0%
Poland	2016	27.2%
Romania	2017	55.1%
Slovenia	2017	42.3%

Ever-use measurement counts a substantial number of youth who experiment with ENDS but do not progress to regular use [53]. Similarly, among youth aged 13 to 17 in Norway in a 4-year longitudinal (2015–2019) qualitative study (50 semi-structured group and 175 individual interviews), it was often the case that as youth became older, ENDS use became viewed as a childish practice that they discarded [54]. The 2015 ESPAD survey did not ask about ever-use of ENDS, so data on trends for most EU member states are not available at this time. The prevalence of ever-use has not increased in Finland since 2015 [55], and ever-use prevalence for 12–18-year-olds in the Netherlands has decreased from 34.1% in 2015 to 25.6% in 2019 [56]—a 25% decrease.

Current youth use, defined as any use in the past 30 days, includes a substantial number of youths who use ENDS infrequently and captures those who tried ENDS only once in their lives, but it happened to be that month. Among EU youth with past-30-day use in the ESPAD 2019 survey [57], half or more reported use less than once a week in 20 of 22 member states. See Table 6.

**Table 6:** Percentage of past-30-day 15–16-year-old users with infrequent use. *Source*: Computed from Table 7a E-Cigarette use during the past 30 days (percentages) [57]

Country	Less than weekly (%)
Austria	59
Croatia	61
Cyprus	50
Denmark	58
Estonia	60
Finland	69
France	68
Germany	70
Greece	65
Hungary	62
Ireland	52
Italy	60
Latvia	59
Lithuania	35
Malta	57
Netherlands	63
Portugal	66
Romania	56
Slovakia	43
Slovenia	62
Spain	69
Sweden	71

Ever-use data for EU adults are also problematic for exposure assessment and estimating prevalence. The 2016 European Regulatory Science on Tobacco (EUREST-PLUS ITC, *N* = 1178) found that among adults reporting ever use in Germany, Greece, Hungary, Poland, Romania, Spain, 38% had used ENDS 1–2 times and 21% had used ENDS 3–10 times. Furthermore, 85% of those who ever used ENDS were no longer using them [58]. Current use of ENDS defined as any-past-30-day use is not a good indicator of sustained use as persons using ENDS 5 days or less a month “includes many individuals who can be expected to discontinue use within 1 year” [59, p. e92].

The classification of ever-use for youth and adults is not a robust measurement of prevalence because many are simply experimenting on a very limited basis and do not continue use. The measurement of any-past-30-day use, the classification for a current use, does not indicate a regular pattern of use.

### Non-nicotine use

The *Opinion* does not include data on the use of non-nicotine ENDS, an omission of important data on nicotine exposure. In the EU, evidence shows that a substantial number of youth and adults do not use nicotine liquids.

Many EU youth reporting ever using ENDS state that that they used non-nicotine products. In Finland, 52% of boys and 48% of girls used only non-nicotine ENDS, and nicotine use declined from 2013 to 2019 [55]. In France, 42.2% of youth who have ever smoked tobacco and 92.9% of those who have never smoked used only non-nicotine ENDS [60]. In Italy, 72.0% overall used non-nicotine liquids, 40.3% used non-nicotine exclusively and 31.7% used both nicotine and non-nicotine [49]. In Sweden, 38% used only non-nicotine ENDS [61]. A 2-year longitudinal cohort study in Finland (*N* = 3474) found that exclusive use of non-nicotine ENDS by adolescents was not associated with a higher probability of daily smoking compared to youth who has never used ENDS [62].

Non-nicotine ENDS use also appears to be common among youth classified as having “current” (recent) ENDS use. A US survey (*N* = 1589, 15–17 years old) of youth reporting past-30-day use found that 29% used only non-nicotine liquids and 39% reported using both nicotine and non-nicotine liquids [63].

Three studies indicate that many EU adults who use ENDS were using non-nicotine liquids. A 2016 survey of French young adults (19–22 years old) with recent use of ENDS found that 61 of 98 used only non-nicotine ENDS and an additional 19 reported using both [64]. A 2016 face-to-face interview project in Barcelona, Spain, with 600 adults using ENDS daily reported that 33.7% who were quitting smoking and 43.6% who had reduced cigarette use did not use nicotine liquids [65]. In a 2016 online survey in Poland of those currently using ENDS (*N* = 1142), 9.8% started ENDS use because they could use non-nicotine liquids [66].

Nicotine use during pregnancy is a known risk factor for adverse neonatal outcomes. A 2015 survey [67] of women in two US states having given birth that year found that 35.2% of women who used ENDS even once in the last 3 months of pregnancy used non-nicotine ENDS. During the period of 3 months before pregnancy to 2–6 months after delivery, 41.4% used only non-nicotine ENDS.

Exposure assessments for ENDS use are complex. Approximately 75% of the *Opinion’s* statements on exposures are based on ever-use data. Infrequent use should, at a minimum, be noted in the exposure assessments. Reporting the prevalence of non-nicotine use is critical to exposure assessments because of its implications for the risks of nicotine addiction, pregnancy outcomes, and for the cardiovascular effects of ENDS use (see section following).

## Incorrect assessment of cardiovascular risk

The *Opinion* states that there is strong evidence for long-term systematic effects of ENDS on the cardiovascular system. This statement on cardiovascular risk is contradicted by numerous researchers and reviews. The National Academies of Sciences, Engineering, and Medicine systematic review [9] concluded that “There is **no available evidence** whether or not e-cigarette use is associated with clinical cardiovascular outcomes (coronary heart disease, stroke, and peripheral artery disease) and subclinical atherosclerosis (carotid intima-media thickness and coronary artery calcification)” (p. 7, emphasis in original). Benowitz and Fraiman [68] and D’Amario et al. [69] stated that there is no available evidence on cardiovascular risk. Münzel et al. [70] in their review concluded that strong evidence on long-term effects was missing.

Two review teams observed that the assessment of cardiovascular risk was controversial and that any risk may be attributed solely to nicotine [10, 71]. This controversy is evidenced in the conflicting results from two clinical studies. A randomized crossover trial (*N* = 25) of nicotine and non-nicotine ENDS use did not find negative changes in micro- and macrovascular endothelial function or oxidative stress with non-nicotine ENDS [72]. This group of researchers concluded that negative cardiovascular effects of ENDS use was attributable to the nicotine present in the liquid. Yet the clinical trial by George et al. [34] discussed earlier found no difference in cardiovascular effects between nicotine and non-nicotine ENDS.

Benowitz and Burbank [73] characterized the cardiovascular risks of ENDS as “very low” for those with no known cardiovascular disease, while those with cardiovascular disease “might incur some increased risk” albeit far lower than smoking (p. 521). They concluded that.

If e-cigarettes can be substituted completely for conventional cigarettes, the harms from smoking would be substantially reduced and there would likely be a substantial net benefit for cardiovascular health (p. 521).

A 4-month randomized observational study divided 40 participants who smoked into an ENDS arm and a continued smoking arm [74]. The participants in the ENDS arm demonstrated reductions in arterial stiffness and oxidative stress that were not experienced by the participants who continued to smoke.

A large US dataset, the National Health Interview Survey, was examined for evidence of ENDS use and cardiovascular risks [75]. A pooled analysis of the 2016 and 2017 surveys did not find an association between ENDS use and myocardial infarction or coronary heart disease.

The *Opinion’s* statement that the overall weight of the evidence of ENDS use for long-term systemic cardiovascular effects is strong is contradicted by the quantity of studies and reviews that stated there is a lack of evidence. Some studies ascribe cardiovascular risk to nicotine, others found no risk, and one RCT demonstrated improvements in cardiovascular function.

## Decontextualizing the role of flavours

While the *Opinion* discusses the role of flavours in attracting youth to ENDS, the most common reason by far for youth and young adult ENDS experimentation is curiosity, not flavours. A 2018 survey of French youth (age 15–16, *N* = 1435) found that curiosity was the most common reason for trying ENDS, followed by flavours [76], data not reported. In a 2016 survey in Germany (*n* = 474) of respondents aged 14 and older who had ever used ENDS 59.5% endorsed curiosity as the reason for trying ENDS, increasing to 73.1% in the 14–19-year-old age-group [77]. For French young adults (19–22 years old), a 2016 survey (*N* = 2720) found that 77.4% tried ENDS out of curiosity, 63.5% because someone offered it to them, and 24.6% because of flavours [64]. In the 2019 US National Youth Tobacco Survey (NYTS) [78] flavours ranked third in the reasons for use (22.3%), with curiosity the major reason for trying ENDS (56.1%), followed by use by family or peers (23.9%).

Interestingly, the ability to use ENDS for performing vape tricks was just as common a reason as flavours for trying ENDS at 21.2% in the 2019 NYTS. Tricks with ENDS have been reported as very popular among US youth. In a 2017 survey (*N* = 2945 high school students), 54.4% of students who had ever used ENDS had done vape tricks [79]. Approximately three out of four US youth who endorsed using ENDS in the past 30 days said they performed vape tricks; in a 2016 survey, the number was 75% (*N* = 1729, aged 15–17) [80] and 73% in another survey [79]. It appears that vape tricks may attract adults too. In a small survey (*n* = 183 respondents using ENDS) conducted in the Netherlands, 24.6% endorsed cloud chasing tricks as an attractive feature of ENDS [81]. Attractive features, such as the generation of very dense aerosols, appear to entice people into using these products and not just flavours.

The use of flavours does not appear to increase the risk of tobacco smoking initiation for youth. A US longitudinal cohort study [82] analysed data from waves 1 to 4 of the Population Assessment of Tobacco and Health (PATH) Study (2013 to 2018, *n* = 7311), and the use of ENDS with nontobacco flavours was less strongly associated with youth smoking initiation than the use of tobacco flavours (AOR 0.66; CI 0.16–2.76).

The use of flavours appears to improve cessation success rates for adults. The PATH longitudinal cohort study noted above [82] also analysed cessation by adults under 55 years old (*n* = 5984), and the use of non-tobacco flavours was more strongly associated with smoking cessation compared to tobacco flavours (2.28; CI 1.04–5.01). This finding is corroborated by the longitudinal data analysis of PATH waves 1 through 3 by another research group [83]. Their analysis broke down the increase in cessation rates by length of cessation, with non-tobacco flavoured ENDS substantially associated with higher cessation rates among US adults using ENDS for smoking cessation, RRR 1.75 (CI 1.18–2.60) for those who stopped tobacco smoking in the past year and RRR 2.83 (CI 1.69–4.73) for those achieving 1 year or longer of cessation.

## Cessation

A large number of respondents in an EU survey reported using ENDS in their smoking quit attempt, averaging about one in five, see Table 7. The use of ENDS for quitting is widespread in France, with 76.3% of those who had quit smoking for at least 1 month, stating that they had used ENDS as a quit aid [2017 Eurobarometer Survey, 84].

**Table 7:** Use of ENDS for a quit attempt, EU member states. *Source*: [13]

Member state	Used ENDS in quit attempt (%)
Germany	15.9
Greece	28.7
Hungary	16.2
Netherlands	43.8
Poland	13.0
Romania	11.0
Spain	5.0

Evidence presented in three reviews points to ENDS as an effective cessation aid for those who are already attempting to quit smoking. The most recent Cochrane review [85], published subsequent to the posting of the *Opinion*, concluded that there is moderate-certainty evidence that ENDS use for cessation results in a higher quit rate than NRT, RR 1.69 (CI 1.25–2.27). ENDS produced a higher quit rate than behavioural support only or no support, RR 2.50 (CI 1.24–5.04), although the evidence was of very low certainty. A systematic review and meta-analysis [86] of 14 studies with 35,665 participants calculated quit rate efficiency from 13.2 to 22.9%. The reviewers characterized ENDS as a “promising” cessation aid. A review [87] in *Pharmacotherapy*, a journal of the American College of Clinical Pharmacy, stated that ENDS “may have modest effects to help tobacco users achieve cessation” in a number of different patient populations (p. 565). In addition to these reviews, longitudinal data from the US Population Assessment of Smoking and Health surveys (PATH, *n* = 9724) showed that people making a quit attempt with ENDS were 1.32 (CI 1.03–1.71) times more likely to quit in the past year than those making a quit attempt without ENDS [83].

At the clinical level, a Belgium case report of ENDS use for cessation by patients in treatment with tobacco counsellors, at 7 months (*n* = 103, 70 using ENDS) almost 40% had biochemical-verified abstinence, RR 1.71 (CI 1.04–2.81) compared to NRT users [88]. ENDS are recommended as cessation help by the UK National Health Services on its website: *Using e-cigarettes to stop smoking.* It states that *“Many thousands of people in the UK have already stopped smoking with the help of an e-cigarette. There's growing evidence that they can be effective”* [89]*.*

## Preliminary opinion references

Of note, we identified 21 articles in the References that were not cited in the text and provided this information to the Committee in our submission. Listing these articles as references without any discussion misrepresents which studies were evaluated or how they were interpreted by the Committee.

## Conclusions

Based on the evidence presented, the following changes in Table 8 are recommended for the final report.

**Table 8:** Recommendations for additions and revisions

Opinion section(s)	Recommendation
6.7	Add studies on cigarette quit intentions and quit success
6.5.3	Add studies on the reduction in emissions of ENDS compared to cigarettes
6.5.4	Add biomarker studies showing ENDS exposures for users compared to cigarettes
6.5.4	Add studies on human health effects of ENDS substitution for cigarettes
None	Add studies on population health effects and economic considerations
3.2 6.5.4 6.6	Discuss alternative hypotheses to gateway for explaining youth co-use of ENDS and tobacco products
6.6	Examine studies that contradict the gateway hypothesis
6.6 6.7	Show evidence of the longitudinal decline of cigarette use in EU member states
6.5.1	Show data demonstrating the stability in the prevalence rates of ENDS use by adults
3.2 6.5.1 6.6	Avoid citing ever-use data as a proxy or indicator for prevalence
6.5.1	Avoid citing past-30-day use as a proxy or indicator for regular use
6.5.2	Cite frequency of use data where available
6.4 6.5.2	Add data on non-nicotine use
6.1 6.5.3 6.5.4	Cite available studies and reviews with conflicting appraisals of cardiovascular risk
3.2 6.5.4 6.6	Contextualize the role of flavours in youth experimentation with the primary motivation of curiosity, and other factors such as vape tricks
3.3 6.7	Substantially enlarge the discussion of ENDS for cessation

The point of this critique is not to denigrate the *Preliminary Opinion*, but to promote a thorough reporting of the data, studies, and reviews on ENDS in the final report. We hope that the literature cited here will prove useful. Our goal is to support the revision of the *Preliminary Opinion* into a more robust and factually accurate final report.

## Data Availability

Data for Tables 1, 2, 3, 6, and 7 were extracted from the published articles cited as the source. Data for Table 4 are from the Passport database and are not publicly available. It is available only by subscription from EUROMONITOR International, London, a privately owned market research company. The company can be contacted at https://www.euromonitor.com/. Data for Table 5 are from the Global Youth Tobacco Surveys with open access at the World Health Organization NCD Microdata Repository: https://extranet.who.int/ncdsmicrodata/index.php/home.

## Abbreviations

BP: Blood pressure
CI 95%: Confidence interval
COPD: Chronic obstructive pulmonary disease
ENDS: Electronic nicotine delivery system(s)
ESPAD: European School Survey Project on Alcohol and Other Drugs
EU: European Union
GYTS: Global Youth Tobacco Survey
N: Total number of participants
n: Subset of participants
NR: Not reported
NYTS: US National Youth Tobacco Survey
PATH: US Population Assessment of Tobacco and Health Survey
RR: Relative risk
RRR: Relative risk ratio
US: United States
WHO: World Health Organization
